# Deep Learning-Based Multi-Class Pediatric Wrist Fracture Subtype Classification: A Pilot Study Comparing Convolutional Neural Network Architectures

**DOI:** 10.3390/jimaging12070307

**Published:** 2026-07-08

**Authors:** Rohan A. Phadke, Samer G. Salman, Zane G. Salman, Sai M. Yedupati, Joshua Ong, Alireza Tavakkoli, Sainyam Galhotra, Ajay Tripuraneni, James Rizkalla

**Affiliations:** 1 School of Medicine, Baylor College of Medicine, Houston, TX 77030, USA; 2 College of Natural Sciences, The University of Texas at Austin, Austin, TX 78712, USA; zgs277@eid.utexas.edu; 3 College of Engineering and Applied Sciences, University of Cincinnati, Cincinnati, OH 45221, USA; 4 Michigan Medicine, University of Michigan, Ann Arbor, MI 48109, USA; 5 Department of Computer Science, University of Nevada, Reno, NV 89557, USA; 6 Department of Computer Science, Cornell University, Ithaca, NY 14853, USA; 7 Department of Orthopaedic Surgery, Baylor University Medical Center, Dallas, TX 75246, USA

**Keywords:** pediatric wrist fractures, distal radius fractures, fracture subtype classification, pediatric radiography, musculoskeletal radiology, artificial intelligence, deep learning, convolutional neural networks, transfer learning, DenseNet, medical image classification, GRAZPEDWRI-DX, Salter–Harris fractures, torus fractures, Grad-CAM

## Abstract

Pediatric wrist fractures are among the most prevalent musculoskeletal injuries in children. Fracture subtype, including buckle/torus, greenstick, and Salter–Harris physeal injuries, directly influences management and prognosis. Subspecialty radiographic expertise required for subtype classification is not universally available in emergency or resource-limited settings. Deep learning (DL) offers an automated approach to fracture subtype recognition from plain radiographs. This pilot study evaluated convolutional neural network (CNN)-based five-class pediatric wrist fracture classification using the GRAZPEDWRI-DX dataset.A total of 940 pediatric wrist radiographs from GRAZPEDWRI-DX (figshare ID 14825193) were labeled using Arbeitsgemeinschaft fur Osteosynthesefragen (AO) pediatric codes into five classes: no fracture, buckle/torus, greenstick, Salter–Harris physeal fracture, and other fracture. Contrast-limited adaptive histogram equalization (CLAHE) and letterbox resizing to 224 × 224 pixels were applied. Patient-level stratified splits (70/15/15%) prevented data leakage. Three ImageNet-pretrained architectures (DenseNet-169, ResNet-50, and EfficientNet-B4) underwent two-phase transfer learning. Performance was assessed by balanced accuracy, macro F1, macro area under the receiver operating characteristic curve (AUROC), and Cohen’s kappa.DenseNet-169 achieved the highest balanced accuracy (0.371; 95% confidence interval [CI]: 0.289–0.448), macro F1 (0.334; 95% CI: 0.251–0.416), and macro AUROC (0.669), with Cohen’s kappa of 0.269 on the held-out test set (*n* = 139) under initial five-epoch pilot training conditions. All three networks exceeded a majority-class (no-information) baseline (balanced accuracy 0.20). Extending training to 50 epochs (approximately 2100 mini-batch iterations) with GPU acceleration substantially improved DenseNet-169 to a balanced accuracy of 0.532 (95% CI: 0.451–0.614), macro F1 of 0.516, and macro AUROC of 0.815, with statistically significant pairwise architecture differences (McNemar *p* < 0.01); per-class sensitivity was highest for no-fracture detection (0.969) and lowest for buckle/torus fractures (0.393). Gradient-weighted class activation mapping (Grad-CAM) confirmed anatomically coherent model saliency at the distal radial metaphysis and physeal plate.DenseNet-169 achieved the best five-class classification performance among evaluated architectures under pilot training conditions, and extended training substantially improved accuracy, although classification accuracy remained below clinically usable thresholds. These results establish a reproducible, patient-stratified DL pipeline and a benchmark for full-dataset training and future methodological development, rather than a clinically deployable tool.

## 1. Introduction

Fractures are common during childhood and adolescence, and the distal forearm is the single most frequently fractured site; population-based data indicate that pediatric fracture incidence has risen over recent decades in parallel with increasing participation in sports and recreational activity [1]. Distal radius fractures are the most common fractures in children, accounting for approximately one-quarter to one-third of all pediatric fractures, with reported annual incidences of 30 per 10,000 children in the United States [2]. Torus fractures are the most prevalent subtype, representing 44% of distal radius fractures in a prospective multicenter registry of 1951 patients, followed by bicortical (31%) and physeal (21%) fractures [3].

The biomechanical properties of immature bone produce fracture patterns unique to the pediatric skeleton, including buckle (torus), greenstick, and Salter–Harris physeal injuries, each carrying distinct implications for management and prognosis [2]. Torus fractures are stable injuries amenable to symptomatic treatment, whereas Salter–Harris physeal fractures risk growth disturbance and may require operative intervention [4,5]. Greenstick and other incomplete fractures occupy an intermediate position, and management decisions, including the need for reduction, the type of immobilization, and the interval to follow-up imaging, are tied directly to the specific subtype rather than to the mere presence of a fracture [2,4].

Accurate subtype classification is therefore essential for appropriate triage, yet it demands subspecialty radiographic expertise that is not universally available. Emergency physician interpretation of pediatric skeletal radiographs carries a misdiagnosis rate of approximately 5–8%, with the wrist among the most error-prone regions, and discordant interpretations lead to increased subsequent healthcare utilization [6,7]. These error rates, together with the limited availability of pediatric radiology expertise in emergency and resource-limited settings, motivate automated decision support able to characterize fracture subtype at the point of care.

Deep learning with convolutional neural networks (CNNs) has emerged as a promising tool for automated fracture detection on plain radiographs [8,9]. Recent meta-analyses of AI-based pediatric fracture detection report pooled sensitivities of 92–93% and specificities of 90–91%, approaching expert-level performance [10,11]. Multicenter reader studies have further demonstrated that AI assistance significantly improves diagnostic accuracy and reduces reading time for non-specialist clinicians [12,13].

Most of these systems are built on established CNN backbones, such as the residual connections of ResNet, the dense connectivity of DenseNet, and the compound scaling of EfficientNet, which are adapted to medical tasks through transfer learning from large natural-image corpora [14,15,16]. Because the backbone is pretrained, transfer learning markedly reduces both the number of parameters that must be estimated de novo and the volume of labeled medical data required to reach a given level of accuracy, a property that is particularly valuable in pediatric imaging where annotated subtype-level data are scarce [17,18]. However, the vast majority of existing models perform binary fracture-versus-no-fracture classification rather than multi-class subtype discrimination, leaving a critical gap between fracture detection and the clinically actionable subtype information that guides treatment decisions.

Beyond fracture detection, artificial intelligence and machine learning are increasingly applied across orthopedic care for perioperative risk prediction and multimodal data integration, although inconsistent external validation and persistent translational barriers continue to limit clinical adoption [19,20,21,22]. Realizing this potential will also depend on building artificial intelligence literacy among orthopedic trainees and clinicians [23].

To date, several studies have applied deep learning to the publicly available GRAZPEDWRI-DX pediatric wrist radiograph dataset for fracture detection and localization using object-detection frameworks such as YOLO, but none have addressed multi-class fracture subtype classification [24,25,26]. Although GRAZPEDWRI-DX has become a widely used benchmark for fracture detection and localization [24,25,26,27], to our knowledge no prior study has repurposed its Arbeitsgemeinschaft fur Osteosynthesefragen (AO) pediatric annotations for multi-class subtype classification; the transition from detecting that a fracture is present to characterizing which subtype it represents therefore remains largely unexplored. This pilot study evaluated three ImageNet-pretrained CNN architectures, DenseNet-169, ResNet-50, and EfficientNet-B4, for five-class pediatric wrist fracture subtype classification (no fracture, buckle/torus, greenstick, Salter–Harris, and other fracture) using a patient-stratified subset of GRAZPEDWRI-DX, establishing a reproducible, patient-stratified deep learning pipeline as a foundation for full-dataset training and subsequent methodological development.

## 2. Methods

### 2.1. Study Design and Dataset

**Figure 1:**
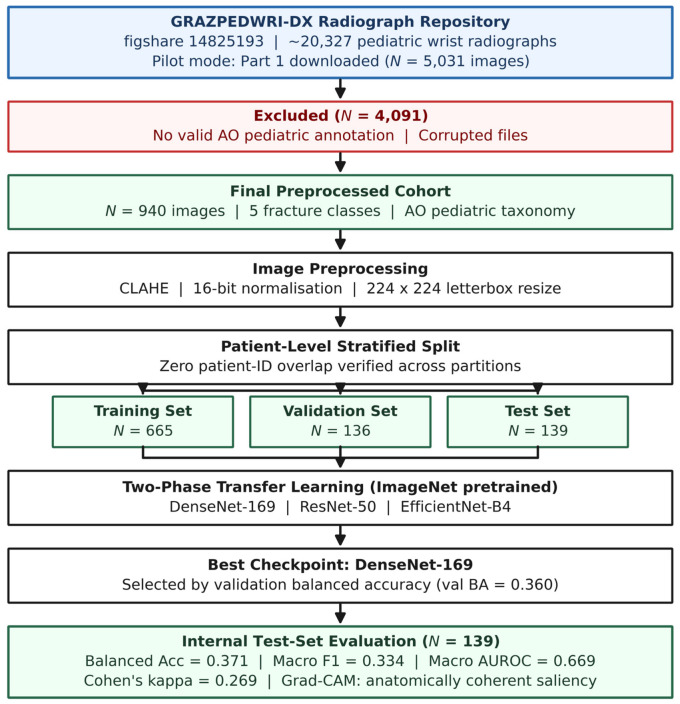
Study Flow Diagram: GRAZPEDWRI-DX Pilot (*n* = 940). Data acquisition, exclusion criteria, preprocessing, patient-level stratified splitting, two-phase transfer learning, and internal test-set evaluation. AO = Arbeitsgemeinschaft fur Osteosynthesefragen; CLAHE = contrast-limited adaptive histogram equalization; AUROC = area under the receiver operating characteristic curve; Grad-CAM = gradient-weighted class activation mapping.

This pilot study used a retrospective observational design with publicly available, de-identified data. Pediatric wrist radiographs were sourced from the GRAZPEDWRI-DX repository (figshare ID 14825193), a multicenter dataset comprising approximately 20,327 anteroposterior and lateral wrist radiographs from patients 18 years and younger. For pilot feasibility analysis, the Part 1 archive was downloaded (*n* = 5031 images). Images were categorized into five fracture classes using Arbeitsgemeinschaft fur Osteosynthesefragen (AO) pediatric codes: no fracture, buckle/torus, greenstick, Salter–Harris physeal fracture, and other fracture. Images lacking valid AO annotations or identified as corrupted were excluded (*n* = 4091), yielding a final cohort of 940 images (Figure 1, Table 1). Exclusions were driven predominantly by the absence of AO pediatric subtype codes in the available Part 1 metadata rather than by image quality, with only a minority attributable to file corruption; because the retained images were therefore those carrying complete subtype annotations, the pilot cohort may not be fully representative of the broader GRAZPEDWRI-DX population, a potential selection bias addressed in the Section 4.1.

### 2.2. Image Preprocessing

All radiographs underwent contrast-limited adaptive histogram equalization (CLAHE; clip limit 2.0, tile grid 8 × 8) to enhance bone and soft tissue contrast. Images were subsequently normalized from 16-bit to 8-bit depth and letterbox-resized to 224 × 224 pixels to preserve native anatomical aspect ratios without distortion. Training augmentation, applied via the albumentations library (v2.0.8), included random horizontal flipping, rotation up to 15 degrees, and brightness and contrast jitter. Complete preprocessing and pipeline configuration parameters are detailed in Table 2.

### 2.3. Data Splitting

To prevent patient-level data leakage, a critical concern in radiographic deep learning studies where a single patient may contribute multiple images, all dataset partitions were constructed using unique patient identifiers rather than individual image indices. The final cohort (*n* = 940) was partitioned into training (70%; *n* = 665), validation (15%; *n* = 136), and test (15%; *n* = 139) sets, with stratification by fracture class to preserve proportional class representation across all partitions. Zero patient-identifier overlap across all three splits was verified programmatically before any model training (Table 1).

### 2.4. Model Architectures and Training

Three CNN architectures were evaluated—DenseNet-169, ResNet-50, and EfficientNet-B4, each initialized with ImageNet-pretrained weights using the timm library (v1.0.27). These architectures represent distinct design paradigms, including dense skip connections (DenseNet-169), residual connections (ResNet-50), and compound scaling (EfficientNet-B4), enabling assessment of whether architectural inductive biases influence fracture subtype discriminability on small pediatric radiograph datasets. A two-phase transfer learning protocol was applied. In Phase 1, only the classification head was trained with the backbone frozen at a learning rate (LR) of 1 × 10^−3^. In Phase 2, end-to-end fine-tuning was performed at LR 1 × 10^−4^, for a total of five pilot epochs. Class-weighted cross-entropy loss was used throughout training to address class imbalance. Best-performing model checkpoints were selected based on peak validation balanced accuracy. This checkpoint-selection rule served as a de facto early-stopping criterion; formal early stopping and GPU acceleration were not employed at pilot scale and are planned for the definitive analysis. Because Phase 1 updated only the classification head and Phase 2 fine-tuned pretrained backbones, the number of parameters estimated de novo was small relative to the total network size, which partially offsets the limited sample size; nonetheless, 940 images remain below common heuristics, relating training-set size to model capacity, further motivating the full-dataset study [17,18]. Pilot experiments were performed using PyTorch (v2.8.0) in a CPU-only pilot environment. Architecture and training parameters are summarized in Table 3. To evaluate whether the brief pilot training duration constrained performance, all three architectures were additionally retrained for 50 epochs (approximately 2100 mini-batch iterations) using identical data splits, preprocessing, and class-weighted loss, with cosine-annealing learning-rate scheduling and GPU (Metal Performance Shaders) acceleration; this extended-training analysis is reported in Section 3.6. Software and hardware used for reproducibility: Python (version 3.14.2), PyTorch (v2.8.0), timm and albumentations (open-source Python packages distributed via PyPI), and, for the extended-training analysis, Apple Metal Performance Shaders GPU acceleration (Apple Inc., Cupertino, CA, USA); no proprietary reagents, devices, or commercial cell lines were used in this computational study.

### 2.5. Statistical Analysis

**Figure 2:**
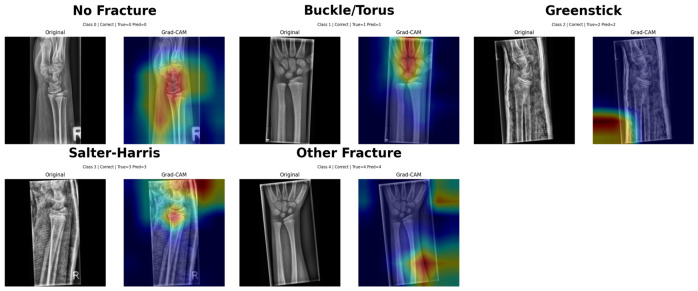
Gradient-weighted class activation mapping (Grad-CAM) saliency: DenseNet-169. Representative activation maps overlaid on correctly classified wrist radiographs for each fracture class. Model attention localizes to the distal radial metaphysis, physeal plate, and cortical margins, consistent with expected radiographic fracture localization. Warmer colors (red/yellow) indicate regions of higher model attention; cooler colors (blue) indicate lower attention.

Primary performance metrics were computed on the held-out test set (*n* = 139) and included balanced accuracy, macro-averaged F1 score, macro AUROC, and Cohen’s kappa. Balanced accuracy was selected as the primary endpoint given class imbalance across fracture subtypes; it is computed as the unweighted mean of per-class recall and is more informative than overall accuracy in imbalanced multi-class settings. Bootstrap confidence intervals (CIs) were generated over 200 stratified resampling iterations to quantify metric variability at the pilot scale. The relatively small number of iterations reflects the pilot scope and CPU-only compute, so the resulting intervals are descriptive; the definitive analysis will employ at least 2000 iterations. Pairwise architecture comparisons used McNemar’s test on binary correct/incorrect prediction vectors. McNemar’s test was applied to the paired correct/incorrect indicator vectors of two models on the shared test set, a standard and appropriate procedure for comparing two classifiers on identical data that does not require the underlying task to be binary [28]; balanced accuracy and macro F1 served as the primary descriptive metrics for the five-class comparison. Grad-CAM was applied to DenseNet-169 to generate class-specific saliency maps and provides a qualitative interpretability assessment of spatial model attention (Figure 2).

## 3. Results

### 3.1. Study Cohort

A total of 940 pediatric wrist radiographs were included in the final analytic cohort after exclusion of 4091 images lacking valid AO pediatric code annotations or identified as corrupted (Figure 1). Class distribution was preserved across training (*n* = 665), validation (*n* = 136), and test (*n* = 139) partitions by stratified design (Table 1). The no-fracture, buckle/torus, greenstick, and Salter–Harris classes each comprised approximately 20–22% of the training set, while the other fracture class represented the smallest proportion (14.4%; *n* = 96 training; 9.4%; *n* = 13 test), reflecting real-world class imbalance within the available pilot archive.

### 3.2. Overall Model Performance

DenseNet-169 achieved the highest balanced accuracy (0.371; 95% CI: 0.289–0.448), macro F1 score (0.334; 95% CI: 0.251–0.416), and Cohen’s kappa (0.269) on the internal test set (*n* = 139), demonstrating the most consistent overall classification performance across all five fracture classes (Table 4). ResNet-50 achieved a marginally higher macro AUROC (0.679) but substantially lower macro F1 (0.150; 95% CI: 0.116–0.188), suggesting collapsed predictions toward a subset of classes. EfficientNet-B4 demonstrated the lowest balanced accuracy (0.299; 95% CI: 0.215–0.384) and macro AUROC (0.640). McNemar’s test revealed no statistically significant pairwise architecture differences, consistent with the limited training scale of this pilot analysis. Notably, all three architectures exceeded a majority-class (no-information) baseline, which predicts the most frequent class for every case and corresponds to a balanced accuracy of 0.200 and a macro AUROC of 0.500; this baseline is included in Table 4 as the minimum reference standard.

### 3.3. Per-Class Performance: DenseNet-169

**Figure 3:**
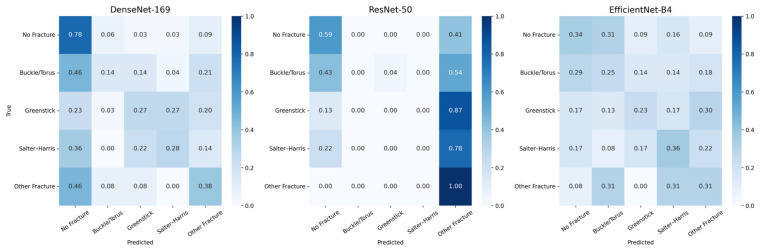
Confusion matrices: DenseNet-169, ResNet-50, and EfficientNet-B4 (Internal Test Set, *n* = 139). Normalized confusion matrices displaying per-class classification rates for each architecture. Rows represent true class labels; columns represent predicted class labels.

**Figure 4:**
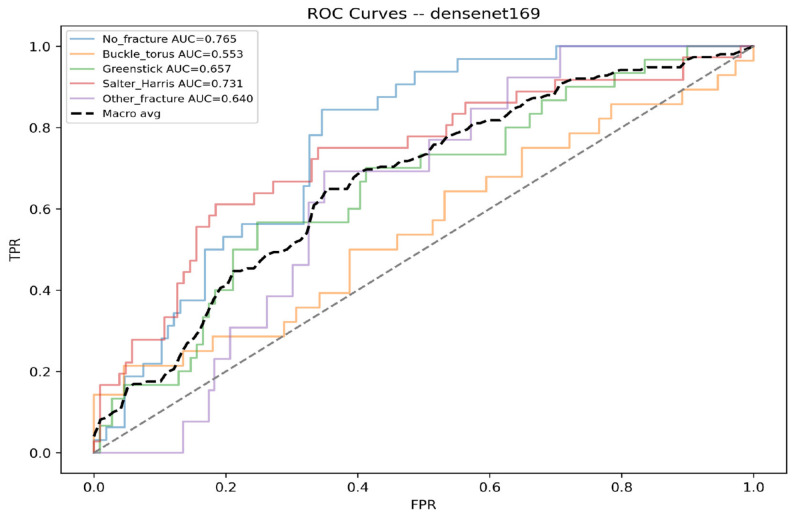
Receiver operating characteristic curves: DenseNet-169 (Best Model, *n* = 139). One-vs-rest ROC curves for each of the five fracture classes under DenseNet-169. AUROC values range from 0.553 (Buckle/Torus) to 0.765 (No Fracture). AUROC = area under the receiver operating characteristic curve.

Per-class performance metrics for DenseNet-169 are presented in Table 5 and illustrated through normalized confusion matrices in Figure 3. The no-fracture class demonstrated the highest sensitivity (0.781) and AUROC (0.765), reflecting reliable identification of radiographically normal wrists. Buckle/torus fractures showed the lowest sensitivity (0.143) alongside the highest class-specific specificity (0.964), indicating a tendency to under-predict this class under limited training data. Salter–Harris physeal fractures achieved the second-highest AUROC (0.731) but modest sensitivity (0.278), consistent with the inherent radiographic subtlety of physeal injuries. Receiver operating characteristic (ROC) curves for each fracture class under DenseNet-169 are presented in Figure 4.

### 3.4. Interpretability: Grad-CAM Saliency

Grad-CAM saliency maps generated from DenseNet-169 are displayed in Figure 2. Grad-CAM computes gradient-weighted activations from the final convolutional layer to produce a class-specific heatmap superimposed on the input radiograph, highlighting regions the model weighted most heavily for each classification decision. In correctly classified fracture cases, model attention was concentrated at anatomically relevant regions, including the distal radial metaphysis, physeal plate, and cortical margins, consistent with expected radiographic localization for each fracture subtype. The spatial coherence of activation maps across fracture subtypes supports the biological plausibility of model predictions at pilot scale and suggests that DenseNet-169 identified genuine fracture-associated imaging features rather than spurious background signals or incidental scanner artifacts.

### 3.5. Training Dynamics

**Figure 5:**
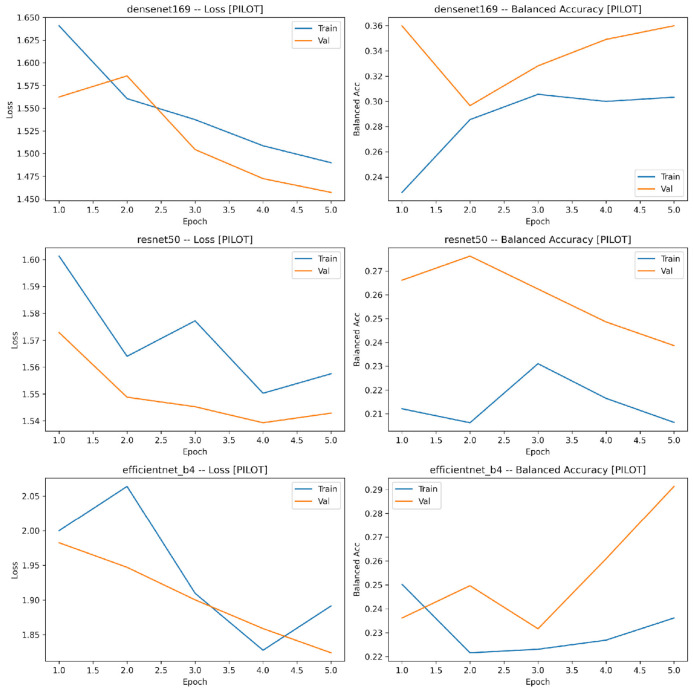
Training and validation curves: All three architectures (pilot, 5 epochs). Loss and balanced accuracy curves for DenseNet-169, ResNet-50, and EfficientNet-B4 across five pilot epochs. DenseNet-169 achieved the highest peak validation balanced accuracy (0.360).

Training and validation loss and balanced accuracy curves are depicted in Figure 5 for all three architectures across five pilot epochs. DenseNet-169 achieved the highest peak validation balanced accuracy (0.360), compared with ResNet-50 (0.276) and EfficientNet-B4 (0.291), consistent with its superior test-set performance. All three architectures displayed progressive training loss reduction across epochs, without evidence of gross divergence or instability, indicating that the two-phase transfer learning protocol was appropriately calibrated for this dataset size. The brevity of pilot training, limited to five epochs on a CPU-only environment, precludes direct performance comparisons to fully trained deep learning models in the published literature; GPU-accelerated training across 30 epochs with the complete GRAZPEDWRI-DX dataset is anticipated for the definitive analysis. Detailed preprocessing and pipeline configuration are provided in Table 2.

### 3.6. Effect of Extended Training

**Figure 6:**
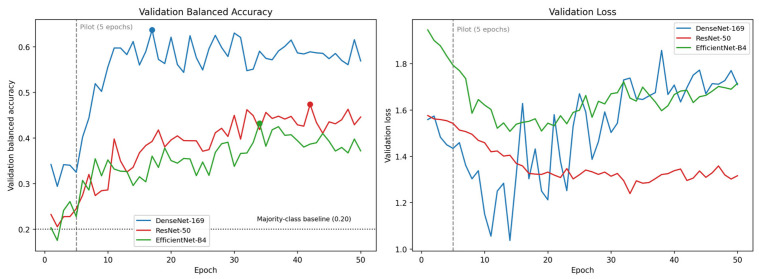
Validation curves under extended training (50 epochs, approximately 2100 mini-batch iterations) for all three architectures. Left: validation balanced accuracy versus epoch, with the five-epoch pilot stopping point and the majority-class baseline (0.20) indicated; filled markers denote each architecture’s peak. Right: validation loss versus epoch. DenseNet-169 reached peak validation balanced accuracy near epoch 17; the subsequent rise in its validation loss reflects mild overfitting on the 940-image cohort and motivates best-checkpoint selection.

All three architectures were retrained under the extended protocol described in Section 2.4. Extending training from five to 50 epochs (approximately 2100 mini-batch iterations) substantially improved performance across architectures (Table 6, Figure 6). DenseNet-169 remained the best-performing model, with balanced accuracy increasing from 0.371 to 0.532 (95% CI: 0.451–0.614), macro F1 from 0.334 to 0.516, macro AUROC from 0.669 to 0.815, and Cohen’s kappa from 0.269 to 0.547. Per-class sensitivity improved most notably for no-fracture detection (0.781 to 0.969) and buckle/torus fractures (0.143 to 0.393), the latter remaining the most challenging subtype. Validation balanced accuracy peaked near epoch 17 and plateaued thereafter (Figure 6), indicating that the five-epoch pilot was substantially undertrained. Under the longer schedule, pairwise architecture differences reached statistical significance: DenseNet-169 outperformed both ResNet-50 (McNemar *p* = 0.007) and EfficientNet-B4 (*p* = 0.001), whereas ResNet-50 and EfficientNet-B4 did not differ significantly (*p* = 0.54). Despite these gains, performance remained below clinically usable thresholds and was obtained on the same 940-image subset, so full-dataset training remains necessary.

## 4. Discussion

This pilot study represents, to our knowledge, the first attempt at multi-class pediatric wrist fracture subtype classification using deep learning on plain radiographs. DenseNet-169 achieved the highest balanced accuracy (0.371), macro F1 (0.334), and Cohen’s kappa (0.269) among the three architectures evaluated, while Grad-CAM saliency maps confirmed anatomically coherent model attention at the distal radial metaphysis and physeal plate. Although these metrics fall below the thresholds required for clinical deployment, they establish proof-of-concept for CNN-based fracture subtype discrimination and identify key challenges that must be addressed in subsequent full-scale training. Beyond demonstrating technical feasibility, the principal contribution of this work is a reproducible, patient-stratified benchmark that repurposes the AO pediatric annotations of GRAZPEDWRI-DX for multi-class subtype classification and quantifies, against a common majority-class reference, the per-class error structure that full-scale training must overcome.

Notably, extending training to 50 epochs (approximately 2100 mini-batch iterations), without any change to the dataset, architecture, or preprocessing, substantially improved performance: DenseNet-169 balanced accuracy rose from 0.371 to 0.532 and macro AUROC from 0.669 to 0.815, and pairwise architecture differences that were non-significant at pilot scale became significant, with DenseNet-169 outperforming both ResNet-50 and EfficientNet-B4 (McNemar *p* < 0.01; Section 3.6, Table 6). This indicates that the initial five-epoch pilot was undertrained rather than fundamentally limited, and that the proposed pipeline reaches markedly higher performance on the same 940-image subset when afforded adequate optimization. The plateau in validation balanced accuracy beyond approximately epoch 17 (Figure 6) further suggests that, at this sample size, additional epochs yield diminishing returns and that the remaining performance gap is more likely attributable to dataset size and class imbalance than to training duration, directly motivating the planned full-dataset study.

The modest overall performance observed in this study should be interpreted in the context of several deliberate pilot-scale constraints. Training was limited to 940 images across five classes over only five epochs in a CPU-only environment, conditions that are substantially more restrictive than those of prior deep learning studies in musculoskeletal imaging, which typically employ thousands to tens of thousands of images with GPU-accelerated training over 30 or more epochs [24,27]. Importantly, the multi-class subtype classification task attempted here is fundamentally more challenging than the binary fracture detection paradigm used in most published work. Recent meta-analyses have reported pooled AUCs of 0.95–0.96 for binary pediatric fracture detection, but these models do not distinguish among fracture subtypes, a clinically essential distinction, as buckle fractures may be managed with simple splinting and early discharge, whereas Salter–Harris injuries require close follow-up to monitor for growth arrest [4,5,10,11].

The superior performance of DenseNet-169 relative to ResNet-50 and EfficientNet-B4 is consistent with findings in other medical imaging domains. In a comparison of CNN architectures for large vessel occlusion detection on CT angiography, DenseNet-121 demonstrated the best generalization on external validation, attributed to its heavy use of residual connections utilizing concatenation, which causes feature maps from earlier layers to be used deeper in the network while aiding gradient flow and regularization [29]. Similarly, in brain tumor classification using transfer learning from ImageNet, DenseNet achieved the highest test accuracy (96%) compared with ResNet (91%), EfficientNet (91%), and MobileNet (93%) [30]. These findings suggest that DenseNet’s dense connectivity pattern, which promotes feature reuse, is particularly advantageous in data-limited medical imaging settings. By contrast, ResNet-50’s additive residual shortcuts may be less effective at preserving fine-grained features critical for distinguishing subtle fracture morphologies. The observation that ResNet-50 achieved a marginally higher macro AUROC (0.679) but substantially lower macro F1 (0.150) suggests class-collapsed predictions, a known failure mode when models trained on imbalanced data converge to predicting the majority class.

Per-class analysis revealed instructive patterns. The no-fracture class achieved the highest sensitivity (0.781) and AUROC (0.765), likely reflecting the more homogeneous radiographic appearance of normal wrists compared with the heterogeneous morphology of fracture subtypes. Buckle/torus fractures demonstrated the lowest sensitivity (0.143), consistent with the well-documented radiographic subtlety of these injuries, which involve only partial cortical compression without a complete break in the cortex [2]. This finding mirrors clinical experience: buckle fractures are among the most frequently missed fracture types by emergency physicians, and their subtle radiographic features present a challenge for both human readers and automated systems [31,32]. Salter–Harris physeal fractures achieved the second-highest AUROC (0.731) but modest sensitivity (0.278), reflecting the inherent difficulty of detecting physeal injuries on plain radiographs, particularly Salter–Harris type I fractures, which involve fracture through the growth plate and may show no visible fracture line on radiographs [5].

The Grad-CAM interpretability analysis provides important qualitative validation. Model attention was concentrated at the distal radial metaphysis and physeal plate regions for correctly classified fracture cases, consistent with the expected anatomic localization of each fracture subtype. This spatial coherence suggests that DenseNet-169 learned genuine fracture-associated imaging features rather than spurious correlations with background artifacts or scanner characteristics, a concern that has been raised in prior analyses of public radiograph datasets [33]. Class activation mapping and related saliency techniques have been increasingly recognized as essential components of trustworthy AI in medical imaging, providing clinicians with visual explanations that support or challenge model predictions [34,35].

### 4.1. Limitations

Several limitations warrant discussion. First, the small sample size (*n* = 940) and brief training duration (five epochs) were intentional pilot constraints designed to validate the data pipeline and patient-level stratification methodology before committing to full-dataset training. The extended-training analysis (Section 3.6) directly addressed this training-duration constraint and markedly improved performance, indicating that sample size and class representativeness, rather than training duration, are now the principal limitations. Second, the class imbalance inherent in the dataset, with the “other fracture” category comprising only 14.4% of training images, likely contributed to reduced performance in minority classes despite the use of class-weighted loss. Third, the use of AO pediatric codes as ground-truth labels, while standardized, introduces dependence on the accuracy of the original annotations in the GRAZPEDWRI-DX dataset. Fourth, this study evaluated only classification architectures; object-detection frameworks such as YOLO, which have shown strong performance for fracture localization on this same dataset, may offer complementary advantages for subtype recognition when combined with classification heads [24,25,26]. Finally, the absence of external validation limits generalizability; future work should incorporate multi-institutional datasets to assess model robustness across different imaging protocols and patient populations.

Several additional constraints temper interpretation of these results. The pilot cohort (*n* = 940) is well below common heuristics relating training-set size to the number of free parameters in deep networks; although transfer learning mitigates this by freezing pretrained backbones and estimating relatively few parameters de novo [17,18], the limited and selectively annotated sample constrains both performance and external validity, and the 940 retained images represent only a fraction of the approximately 20,327-image full dataset. Training in the initial pilot was restricted to five epochs on CPU without formal early stopping, and checkpoint selection by peak validation balanced accuracy provided only an approximate stopping rule, so model convergence was incomplete in the initial pilot, although the extended-training analysis (Section 3.6) demonstrates that longer optimization reaches a validation plateau on this cohort. This study also employed established transfer-learning architectures rather than novel network designs, loss functions, or training strategies; its contribution is a reproducible benchmark and pipeline rather than a methodological innovation, and subsequent work will explore task-specific architectures, imbalance-aware losses such as focal loss, and emerging paradigms including vision–language and knowledge-distillation models [36,37]. With respect to evaluation, only a majority-class baseline was available as a minimum reference standard; traditional machine-learning and trained-from-scratch CNN baselines were beyond the scope of this pilot and are planned for the definitive study. Lastly, the bootstrap confidence intervals were derived from a modest number of resampling iterations and the pairwise McNemar comparisons reflect the limited pilot sample, so all interval estimates and significance tests should be regarded as descriptive rather than confirmatory.

### 4.2. Clinical Implications

The clinical implications of automated fracture subtype classification extend beyond diagnostic accuracy. In emergency departments and resource-limited settings where subspecialty radiographic expertise is unavailable, an AI tool capable of distinguishing fracture subtypes could support triage decisions, for example, flagging Salter–Harris injuries for urgent orthopedic consultation while identifying buckle fractures suitable for conservative management and early discharge [4,38]. Such subtype-aware triage could complement bedside prediction tools that forecast downstream resource needs after orthopedic trauma, supporting earlier discharge planning and more efficient resource allocation [39]. More broadly, accurate diagnostic characterization underpins treatment selection and outcome optimization across orthopedic and spine surgical care, where operative strategy, cost-effectiveness, and complication risk vary substantially by patient and injury factors [40,41,42,43]. The planned definitive analysis, using the complete GRAZPEDWRI-DX dataset with GPU-accelerated training over 30 epochs, is expected to substantially improve classification performance and will incorporate additional strategies to address class imbalance, including synthetic oversampling and mixup augmentation. Ensemble approaches combining multiple architectures may further enhance discriminative performance, as demonstrated in musculoskeletal abnormality detection on the MURA dataset [34]. Emerging methodological paradigms may help to close the performance gap observed here, including vision–language models that jointly learn from radiographs and report text [36], knowledge distillation and teacher–student training that transfers representations from larger teacher networks to data-efficient student models [37], and privacy-preserving federated learning that enables multi-institutional training without centralizing patient data [44].

## 5. Conclusions

In conclusion, this pilot study establishes a reproducible, patient-stratified deep learning pipeline for five-class pediatric wrist fracture subtype classification and identifies DenseNet-169 as the most promising architecture for this task. Extended training (50 epochs, approximately 2100 iterations) substantially improved DenseNet-169 performance (balanced accuracy 0.532; macro AUROC 0.815), confirming that adequate optimization, alongside a larger and more balanced dataset, will be central to closing the remaining performance gap. While current performance is insufficient for clinical application, the anatomically coherent Grad-CAM saliency patterns and the systematic identification of class-specific challenges provide a reproducible methodological foundation and benchmark for full-scale model development, the eventual clinical utility of which remains to be established.

## Figures and Tables

**Table 1:** Dataset characteristics by fracture class and data split.

Fracture Class	Train (n)	Train (%)	Val (n)	Val (%)	Test (n)	Test (%)
No Fracture	140	21.1	28	20.6	32	23.0
Buckle/Torus	146	22.0	26	19.1	28	20.1
Greenstick	142	21.4	28	20.6	30	21.6
Salter–Harris	141	21.2	23	16.9	36	25.9
Other Fracture	96	14.4	31	22.8	13	9.4
Total	665	N/A	136	N/A	139	N/A

*Patient-level stratified splitting applied. Percentages reflect the within-split class proportion. Val = validation. Complete fracture subtype (AO M/4.x) absent from pilot archive.*

**Table 2:** Preprocessing and technical pipeline configuration.

Parameter	Value
Input Image Resolution	224 × 224 pixels
Preprocessing	CLAHE (clip limit 2.0, tile 8 × 8) + letterbox padding
Training Batch Size	16
Loss Function	Class-weighted cross-entropy
Augmentation Library	albumentations v2.0.8
DL Framework	PyTorch v2.8.0
Bootstrap CI Iterations	200 (stratified resampling)
Statistical Tests	McNemar’s test (pairwise architecture comparison)
Saliency Method	Grad-CAM (gradient-weighted class activation mapping)
Compute Environment	CPU-only (pilot mode)

*CLAHE = contrast-limited adaptive histogram equalization; DL = deep learning; CI = confidence interval.*

**Table 3:** Model architecture and training configuration.

Architecture	Approx. Parameters	Phase 1 LR	Phase 2 LR	Pilot Epochs	Val Bal Acc
DenseNet-169	~14 M	1 × 10^−3^	1 × 10^−4^	5	0.360
ResNet-50	~25 M	1 × 10^−3^	1 × 10^−4^	5	0.276
EfficientNet-B4	~19 M	1 × 10^−3^	1 × 10^−4^	5	0.291

*All architectures are ImageNet-pretrained (timm v1.0.27). Phase 1 = frozen backbone; Phase 2 = full end-to-end fine-tuning with lower LR. LR = learning rate; Val Bal Acc = validation balanced accuracy.*

**Table 4:** Internal test-set performance comparison (*n* = 139).

Architecture	Balanced Acc (95% CI)	Macro F1 (95% CI)	Macro AUROC	Cohen’s Kappa	Macro Sensitivity	Macro Specificity
DenseNet-169	0.371 (0.289–0.448)	0.334 (0.251–0.416)	0.669	0.269	0.371	0.843
ResNet-50	0.319 (0.293–0.358)	0.150 (0.116–0.188)	0.679	0.315	0.319	0.823
EfficientNet-B4	0.299 (0.215–0.384)	0.292 (0.210–0.364)	0.640	0.283	0.299	0.826
Majority-class baseline	0.200	0.082	0.500	0.000	0.200	0.800

*Bootstrap 95% CI computed over 200 iterations with stratified resampling. AUROC = area under the receiver operating characteristic curve; CI = confidence interval. The majority-class (no-information) baseline predicts the most frequent test-set class for all cases and is reported as the minimum reference standard.*

**Table 5:** Per-class performance: DenseNet-169 (best architecture).

Fracture Class	Test (n)	Precision	Sensitivity	Specificity	F1 Score	AUROC
No Fracture	32	0.391	0.781	0.636	0.521	0.765
Buckle/Torus	28	0.500	0.143	0.964	0.222	0.553
Greenstick	30	0.364	0.267	0.872	0.308	0.657
Salter–Harris	36	0.500	0.278	0.903	0.357	0.731
Other Fracture	13	0.200	0.385	0.841	0.263	0.640

*Metrics computed on internal test set (n = 139). Sensitivity = recall for the given class. Specificity = one-vs-rest true-negative rate. AUROC = area under the receiver operating characteristic curve.*

**Table 6:** Internal test-set performance after extended training (50 epochs, ~2100 iterations) (*n* = 139).

Architecture	Balanced Acc (95% CI)	Macro F1 (95% CI)	Macro AUROC	Cohen’s Kappa	Macro Sensitivity	Macro Specificity
DenseNet-169	0.532 (0.451–0.614)	0.516 (0.423–0.596)	0.815	0.547	0.532	0.885
ResNet-50	0.439 (0.362–0.524)	0.404 (0.330–0.484)	0.772	0.392	0.439	0.856
EfficientNet-B4	0.348 (0.276–0.425)	0.349 (0.278–0.420)	0.744	0.191	0.348	0.846
Majority-class baseline	0.200	0.082	0.500	0.000	0.200	0.800

Extended training comprised 50 epochs (approximately 2100 mini-batch iterations) per architecture with GPU (metal performance shaders) acceleration, using data splits, preprocessing, and class-weighted loss identical to the pilot. Bootstrap 95% CI computed over 1000 iterations with stratified resampling. The majority-class (no-information) baseline is reproduced from Table 4 for reference. AUROC = area under the receiver operating characteristic curve; CI = confidence interval.

## Data Availability

Publicly available datasets were analyzed in this study. The GRAZPEDWRI-DX dataset analyzed here is openly available via figshare at https://doi.org/10.6084/m9.figshare.14825193, figshare record ID 14825193 (accessed on 15 May 2026).

## References

[1] Hedström E.M., Svensson O., Bergström U., Michno P. (2010). Epidemiology of fractures in children and adolescents. Acta Orthop..

[2] Handoll H.H., Elliott J., Iheozor-Ejiofor Z., Hunter J., Karantana A. (2018). Interventions for treating wrist fractures in children. Cochrane Database Syst. Rev..

[3] Shah A.S., Guzek R.H., Miller M.L., Willey M.C., Mahan S.T., Bae D.S. (2023). Descriptive epidemiology of isolated distal radius fractures in children: Results from a prospective multicenter registry. J. Pediatr. Orthop..

[4] Perry D.C., Achten J., Knight R., Appelbe D., Dutton S.J., Dritsaki M., Mason J.M., Roland D.T., Messahel S., Widnall J. (2022). Immobilisation of torus fractures of the wrist in children (FORCE): A randomised controlled equivalence trial in the UK. Lancet.

[5] Brown J.H., DeLuca S.A. (1992). Growth plate injuries: Salter-Harris classification. Am. Fam. Physician.

[6] Kargl S., Pumberger W., Luczynski S., Moritz T. (2019). Assessment of interpretation of paediatric skeletal radiographs in the emergency room. Clin. Radiol..

[7] Al-Sani F., Prasad S., Panwar J., Stimec J., Khosroawshahi A., Mizzi T., Camp M., Colaco K., Kramer A., Boutis K. (2020). Adverse events from emergency physician pediatric extremity radiograph interpretations: A prospective cohort study. Acad. Emerg. Med..

[8] Bousson V., Benoist N., Guetat P., Attané G., Salvat C., Perronne L. (2023). Application of artificial intelligence to imaging interpretations in the musculoskeletal area: Where are we? Where are we going?. Jt. Bone Spine.

[9] Chea P., Mandell J.C. (2020). Current applications and future directions of deep learning in musculoskeletal radiology. Skelet. Radiol..

[10] Pervez A., Hasan S.U., Norrish A.R. (2026). Convolutional neural networks in paediatric fracture detection: Pooled evidence from a systematic review and meta-analysis. Eur. Radiol..

[11] Ximenes G.F., Costa Á.L., Leite L.L., Costa L.L., Ribeiro M.O., Colares P.G.B., Cerqueira G.S. (2025). Are artificial intelligence models reliable for clinical application in pediatric fracture detection on radiographs? A systematic review and meta-analysis. Clin. Orthop. Relat. Res..

[12] Raj S., Sadegi B., Simon J. (2026). Enhancing pediatric fracture detection: Multicenter evaluation of a deep learning AI model and its impact on radiologist performance. Acad. Radiol..

[13] Gasmi I., Calinghen A., Parienti J.-J., Belloy F., Fohlen A., Pelage J.-P. (2023). Comparison of diagnostic performance of a deep learning algorithm, emergency physicians, junior radiologists and senior radiologists in the detection of appendicular fractures in children. Pediatr. Radiol..

[14] Huang G., Liu Z., Van Der Maaten L., Weinberger K.Q. Densely connected convolutional networks. Proceedings of the 2017 IEEE Conference on Computer Vision and Pattern Recognition (CVPR).

[15] He K., Zhang X., Ren S., Sun J. Deep Residual Learning for Image Recognition. Proceedings of the IEEE Conference on Computer Vision and Pattern Recognition (CVPR).

[16] Tan M., Le Q.V. (2019). EfficientNet: Rethinking model scaling for convolutional neural networks. Proceedings of the 36th Interna-tional Conference on Machine Learning (ICML), Long Beach, CA, USA, 9–15 June 2019.

[17] Tajbakhsh N., Shin J.Y., Gurudu S.R., Hurst R.T., Kendall C.B., Gotway M.B., Liang J. (2016). Convolutional neural networks for medical image analysis: Full training or fine tuning?. IEEE Trans. Med. Imaging.

[18] Cho J., Lee K., Shin E., Choy G., Do S. (2015). How much data is needed to train a medical image deep learning system to achieve nec-essary high accuracy?. arXiv.

[19] Salman S., Phadke R., Kumar R., Momin A., Tavakkoli A. (2026). Risk prediction in spine surgery: A scoping review of traditional models, artificial intelligence, and the challenge of clinical translation. Spine Deform..

[20] Salman S.G., Phadke R., Kumar R., Momin A., Tavakkoli A. (2026). Response to: Comment on ‘Risk prediction in spine surgery: A scoping review of traditional models, artificial intelligence, and the challenge of clinical translation’. Spine Deform..

[21] Salman S.G., Phadke R., Kumar R., Zaman N., Tavakkoli A. (2026). Digital twins and multimodal artificial intelligence in spine care: A scoping review of concepts, evidence, and translational barriers. Spine Deform..

[22] Salman S.G., Phadke R.A., Kumar R., Zaman N., Tavakkoli A. (2026). Response to: Comment on ‘digital twins and multimodal artificial intelligence in spine care: A scoping review of concepts, evidence, and translational barriers’. Spine Deform..

[23] Kumar R., Phadke R., Salman S. (2025). Advancing AI literacy in Canadian orthopedic education: A framework for equitable and inclusive training. Can. Med. Educ. J..

[24] Ju R.-Y., Cai W. (2023). Fracture detection in pediatric wrist trauma X-ray images using YOLOv8 algorithm. Sci. Rep..

[25] Liu D., Yang Z., Bao C., Meng Q. (2025). Artificial intelligence-based method for detecting wrist fractures in children. Sci. Rep..

[26] Li M., Jiang Y., Xu T., Ji P., Hu J., Li X., Liu W., Guo R. (2026). MFSD-YOLO: A multi-scale feature detection network for pediatric wrist abnormalities in radiographic images. PLoS ONE.

[27] Zech J.R., Carotenuto G., Igbinoba Z., Tran C.V., Insley E., Baccarella A., Wong T.T. (2023). Detecting pediatric wrist fractures using deep-learning-based object detection. Pediatr. Radiol..

[28] Dietterich T.G. (1998). Approximate Statistical Tests for Comparing Supervised Classification Learning Algorithms. Neural Comput..

[29] Remedios L.W., Lingam S., Remedios S.W., Gao R., Clark S.W., Davis L.T., Landman B.A. (2021). Comparison of convolutional neural networks for detecting large vessel occlusion on computed tomography angiography. Med. Phys..

[30] Aziz N., Minallah N., Frnda J., Sher M., Zeeshan M., Durrani A.H. (2024). Precision meets generalization: Enhancing brain tumor classification via pretrained DenseNet with global average pooling and hyperparameter tuning. PLoS ONE.

[31] George M.P., Bixby S. (2019). Frequently Missed Fractures in Pediatric Trauma. Radiol. Clin. N. Am..

[32] Li W., Stimec J., Camp M., Pusic M., Herman J., Boutis K. (2022). Pediatric musculoskeletal radiographs: Anatomy and fractures prone to diagnostic error among emergency physicians. J. Emerg. Med..

[33] Oakden-Rayner L. (2020). Exploring large-scale public medical image datasets. Acad. Radiol..

[34] He M., Wang X., Zhao Y. (2021). A calibrated deep learning ensemble for abnormality detection in musculoskeletal radiographs. Sci. Rep..

[35] Ananda A., Ngan K.H., Karabağ C., Ter-Sarkisov A., Alonso E., Reyes-Aldasoro C.C. (2021). Classification and visualisation of normal and abnormal radiographs: A comparison between eleven convolutional neural network architectures. Sensors.

[36] Li X., Li L., Jiang Y., Wang H., Qiao X., Feng T., Luo H., Zhao Y. (2025). Vision-language models in medical image analysis: From simple fusion to general large models. Inf. Fusion.

[37] Li X., Li L., Li M., Yan P., Feng T., Luo H., Zhao Y., Yin S. (2026). Knowledge distillation and teacher–student learning in medical imaging: Comprehensive overview, pivotal role, and future directions. Med. Image Anal..

[38] Ziegner M., Pape J., Lacher M., Brandau A., Kelety T., Mayer S., Hirsch F.W., Rosolowski M., Gräfe D. (2025). Real-life benefit of artificial intelligence-based fracture detection in a pediatric emergency department. Eur. Radiol..

[39] Salman S.G., Phadke R., Carlin T., Rana A., Dawson J.R., Fitzgerald C.A., Seger C.P., Zielinski M.D., Dumas R.P. (2026). The fracture orthopedic risk of non-home discharge (FORD) score: A novel bedside predictive tool for non-home discharge in orthopedic trauma patients. Injury.

[40] Phadke R., Ilyas M.H., Kumar R., Sangineni P., Paidisetty V., Kaur H., Dawson J. (2026). Preoperative malnutrition is associated with increased early complications and long-term mortality following pilon fracture ORIF: A retrospective cohort study. J. Orthop. Rep..

[41] Bouras A., Adio A.A., Kumar R., Phadke R., Rice S.W., Mody K., Ndu A. (2026). Primary tibiotalocalcaneal nailing vs open reduction and internal fixation for fragility ankle fractures in older adults: A Markov model. Foot Ankle Orthop..

[42] Phadke R., Salman S., Kumar R., Paidisetty V., Matthews B., Srinivas R., Vaja S., Lee N.J. (2026). Endoscopic and percutaneous minimally invasive repair of pars interarticularis defects: A systematic review of clinical outcomes. Spine Deform..

[43] Srinivas R., Phadke R., Salman S., Hazem D., Kaur H., Kumar R., Vaja S., Lee N.J. (2026). Endoscopic versus open lumbar decompression: A retrospective cohort study of 31,000 patients with 90-day follow-up. Neurosurg. Rev..

[44] Rieke N., Hancox J., Li W., Milletarì F., Roth H.R., Albarqouni S., Bakas S., Galtier M.N., Landman B.A., Maier-Hein K. (2020). The future of digital health with federated learning. npj Digit. Med..

